# Effects of the culture media optimization on pectinase production by *Enterobacter* sp. PSTB-1

**DOI:** 10.1007/s13205-016-0502-y

**Published:** 2016-09-26

**Authors:** M. Purna chandra Reddy, K. V. Saritha

**Affiliations:** 1 Department of Biotechnology, Sri Venkateswara University, Tirupati, 517 502 India; 2 Department of Future Studies, SVU College of Sciences, Sri Venkateswara University, Tirupati, 517 502 A.P. India

**Keywords:** Mango fruit processing industrial waste (MIW) powder, Pectinase, Submerged fermentation, *Enterobacter* sp. PSTB-1, Response Surface Methodology (RSM), Central composite design (CCD) model

## Abstract

In the present study, media composition for high production of pectinase by *Enterobacter* sp. PSTB-1 in submerged fermentation was optimized using response surface methodology (RSM). Mango fruit processing industrial waste (MIW) was used as substrate (carbon source) as it contains high amount of pectin. *Enterobacter* sp. PSTB-1 used in present study has given pectin clear zone (PCZ) of 34 mm is higher than other isolates. The experimental results made by statistical design for high pectinase production revealed that the suitable media components: NaNO_3_ 2.0 g/l, KCl 0.50 g/l, KH_2_PO_4_ 1.0 g/l, MgSO_4_·7H_2_O 0.50 g/l, Yeast extract 1.0 g/l, mango industrial waste powder 5.0 g/l. The actual pectinase activity was 75.23 % correlated with the predicted pectinase activity where the model (CCD) was significant. Response surface modelling applied effectively to optimize the production of pectinase in submerged fermentation to make the process low cost-effective by using powdered mango industrial waste as substrate.

## Introduction

Mango is the most delicious fruit with its health benefits and commercially valuable seasonal fruit. So, there must be a need to process this fruit to make available its products in all seasons (Indian Horticulture Database 2011; Purnachandra Reddy and Saritha 2013). Many mango fruit processing industries were established throughout the world and were generating huge amounts of waste or residues while processing the mango fruits. It has been reported that 28 to 43 % of the total of mangoes remain as residues mainly constituted by peel and seeds are discarded during the process (Guzman et al. 2013). The mango industrial waste can be used in so many ways such as animal feed, fuel wood (dried seeds) and it can also be useful for vermicompost preparation and biogas production. Mango industrial waste is the best source for the production of industrially important pectinase, as it contains high amounts of pectin in ripened mango (Yashoda et al. 2005). Pectin is a jelly like matrix structural polysaccharide found in the primary cell wall and the middle lamella of fruits and vegetables (Namasivayam et al. 2011). Along with pectin other polysaccharides such as cellulose and xylan type polysaccharides strengthens the structure of cell-walls in the flesh of fruits (Beatriz and Fabrice 2012). Pectinase is mainly useful for increasing the filtration efficiency and clarification of fruit juices, most of these are microbial derived. Most of the Bacterial isolates such as *Bacillus* sp and *Pseudomonas.*sp., *Bacillus* sp*. MFW7, Bacillus cereus*, *Bacillus licheniformis, Bacillus cereus,* and *Staphylococcus aureus* were reported as good pectinase producers (Geetha et al. 2012; Mukesh kumar et al. 2012; Venkata Naga Raju et al. 2013). In the present study dried mango fruit processing industrial waste (MIW powder; one of the polluting solid wastes) has been utilized for the production of pectinase through submerged fermentation by using pectinolytic *Enterobacter* sp. PSTB-1 under RSM conditions.

## Materials and methods

### Sample collection

Fresh, dried and decomposed mango industrial waste samples were collected in sterile polythene covers from different mango fruit processing industries around Chittoor, A.P., India. The collected samples were stored at 4 °C for future purposes.

### Isolation and identification of bacterial strains

The bacterial strains were isolated by serial dilution of 1.0 g of mango industrial waste (mixture of fresh, dried and decomposed) (Aneja 2003). Pure cultures of isolated strains were maintained at 4 °C on nutrient agar medium. These strains were identified by Gram’s staining, biochemical testing (Srivastava 2003).

### Screening for pectinolytic bacterial strains

#### Pectinase production medium

This medium was prepared according to Reda Bayoumi et al. (2008), consists of part (A) and part (B). Part (A) contained (g/l): NaNO_3_ 2.0, KH_2_PO_4_ 1.0, KCl 0.5, MgSO_4_·7H_2_O 0.5, Yeast extract 1.0. These contents were dissolved in 40 ml distilled water and pH adjusted to 7.0 by 5 % NaOH (w/v). Part (B) contained (g/l): MIW powder 5.0, dissolved in 10 ml of distilled water. The two parts (A) and (B) were mixed and sterilized. This medium was inoculated with isolated bacterial strains and incubated at 37 °C for 96 hours, then assayed for pectinase activity.

### Assay medium

The medium composition was (g/l): Peptone 0.5, Beef extracts 0.3, NaCl 0.5, Agar 15, Pectin 4.0, these contents dissolved in distilled water (pH 7.0).

#### Pectinase activity assay

Plates of the same size were poured with equal amounts of sterilized assay medium. After cooling, three wells were made in each plate with sterilized cork borer. Each well was inoculated with 0.1 ml of culture filtrate of (pectinolytic enzyme) production medium. These plates were incubated at 37 °C for two to four days. Then the plates were flooded with hexadecyl trimethyl ammonium bromide (HTAB) solution, clearing zones of the medium was investigated and taken as the criteria for determining the pectinase productivity.

### Species identification of PSTB-1 strain

The species identification of highest pectinase producing PSTB-1 was done by sequencing the 16S r-RNA gene using a suitable primer set; B27 (F); 5′-AGA GTT TGA TCM TGG CTC AG-3′ 1492 (R); 5′-GGT TAC CTT GTT ACG ACT T -3′ by following Alexander Probst et al. (Alexander et al. 2010).

#### PCR

A 14 µl PCR master mix contains (µl); 10X PCR buffer 1.25, MgCl_2_ 0.4, dNTPs 1.0, F-primer 1.0, R-primer 1.0, trehalose 8.25, taq 0.1 and 1.0 µl of template DNA. The PCR was carried out in three steps by 37 cycles. First step is de-naturation (1 cycle) at 94 °C for 3 minutes, second step is annealing (35 cycles) at 50 °C for 33 minutes and the third step is re-naturation (1 cycle) at 72 °C for 10 minutes.

### Optimization of media composition

#### Experimental bergy

Central composite design (Design-Expert 9.0.1 2013) model of RSM for six factors with three replicates has been used in the investigation. The variables used were NaNO_3_ (X_1_), KH_2_PO_4_ (X_2_), KCl (X_3_), MgSO_4_·7H_2_O (X_4_), yeast extract (X_5_) and MIW powder (X_6_) each at low (−1) and high (+1) of coded levels.

#### Preparation of substrate

The collected fresh mango industrial waste was washed and chopped into small pieces and oven dried at 55 °C for 72 hours. The dried waste was ground and sieved through 2.0 mm mesh size and dissolved in water (1:5) and left for overnight (Veeranjaneya Reddy et al. 2011). The liquid containing substrate was extracted with the help of cheese cloth by squeezing. This was used as a carbon source for pectinase production.

#### Preparation of inoculum

The inoculum was prepared by using overnight incubated nutrient broth culture of *Enterobacter* sp. PSTB-1.

#### Pectinase production medium

Mango peel basal medium (MPBM) was used as production medium. The media contents were dissolved in citrate phosphate buffer at pH 6.0 according to design model and sterilised. Then the medium was inoculated with *Enterobacter* sp. PSTB-1 and incubated at 37 °C for 48 hours. The culture filtrate of this medium will be used as enzyme source.

#### Pectinase assay

Pectinase activity assay was done by 3,5-dinitrosalicylic acid (DNS) method (Miller 1959). 0.5 ml of the culture filtrate was used as crude enzyme source; 0.5 ml of 1 % pectin was used as substrate. One unit of the enzyme is equal to 1 U of reducing sugars released, measured in terms of D-Galacturonic acid, produced as a result of enzyme reaction.

#### Statistical analysis

Experimental data was analysed by analysis of variance (ANOVA) according to response surface regression procedure to fit the following second order polynomial Eq. (1) was used to describe the effect of variables in terms of linear (A_1_–A_6_), cross product terms (A_7_–A_21_) and exponents (A_22_–A_27_) using Central Composite Design model.

1 $$ \begin{aligned} Y\,\, = \,\,A_{0} + A_{1} X_{1} + A_{2} X_{2} + A_{3} X_{3} + A_{4} X_{4} + A_{5} X_{5} + A_{6} X_{6} + A_{7} X_{1} X_{2} + A_{8} X_{1} X_{3} \hfill \\ + A_{9} X_{1} X_{4} + A_{10} X_{1} X_{5} + A_{11} X_{1} X_{6} + A_{12} X_{2} X_{3} + A_{13} X_{2} X_{4} + A_{14} X_{2} X_{5} + A_{15} X_{2} X_{6} \hfill \\ + A_{16} X_{3} X_{4} + A_{17} X_{3} X_{5} + A_{18} X_{3} X_{6} + A_{19} X_{4} X_{5} + A_{20} X_{4} X_{6} + A_{21} X_{5} X_{6} + A_{22} X_{1}^{2} \hfill \\ + A_{23} X_{2}^{2} + A_{24} X_{3}^{2} + A_{25} X_{4}^{2} + A_{26} X_{5}^{2} + A_{27} X_{6}^{2} . \hfill \\ \end{aligned} $$

where ‘*Y*’ is the predicted response (Pectinase yield U/ml) and X_1_, X_2_, X_3_, X_4_, X_5_,and X_6_ are the coded values of ingredients NaNO_3_, KH_2_PO_4_, KCl, MgSO_4_·7H_2_O, yeast extract and MIW powder of culture medium, respectively.

## Results and discussion

### Sample collection

There are about 13 mango industries in Chittoor district, A.P., India producing high amount of waste which had been leading to environmental pollution. Thirty samples were collected from ten different mango fruit processing industries around Chittoor. Similarly, the wastes such as orange peel, citrus peel, potato peel, corn tegument, wheat bran and banana peels reported as a source for pectinolytic microorganisms (Geetha et al. 2012).

### Isolation and identification of bacterial strains

Microorganisms are said to be the major bio-transforming agents for their ability to degrade waste. So, the bacterial strains used in the present study were isolated from collected mango industrial waste as they already existed in that particular environment and having the capacity to degrade it. Colony-forming units of bacteria in the different habitats were significantly different. Eight bacterial strains belonging to genus *Bacillus* and *Cocci* were identified based on their Gram’s staining and biochemical characterization. They were given by isolate names such as PSTB-1, PSTB-2, PSTB-3, PSTB-4, PSTB-5, PSTB-6, PSTB-7, and PSTB-8.

### Screening for pectinolytic bacteria

All the isolates (PSTB-1 to PSTB-8) were considered as good pectinase producers with their PCZ value of 34, 26, 22, 15, 18, 16, 18, and 18 mm. Only one strain that is PSTB-1 has been selected for high pectinase production due to its high PCZ value of 34 mm. The PCZ value of *Enterobacter* sp. PSTB1 was similar with that of three bacterial isolates (numbers 4071, 107 and 10104) PCZ value of 32, 34 and 34 mm, respectively by using *Solanum tuberosum* peels as substrate (Reda Bayoumi et al. 2008).

### Species identification of PSTB-1 strain

**Fig. 1:**
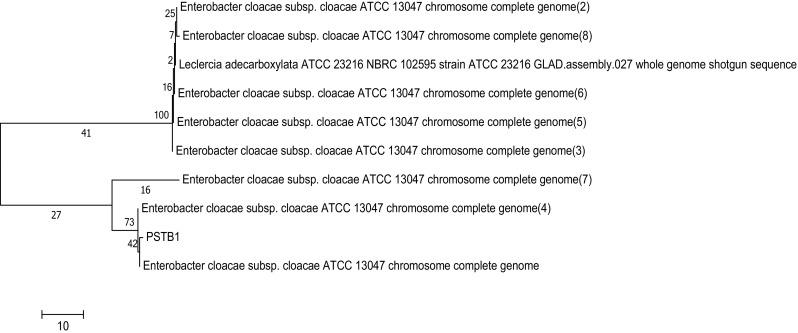
Phylogenetic analysis based on 16S r-RNA sequence available from bacterial isolates was constructed. Evolutionary distances were computed using the p-distance method clustering with the neighbour-joining method

The 16S r-RNA sequence of isolated bacterial strain PSTB-1 has shown more homology with reported strain *Enterobacter cloacae* sub sp. *Cloacae* (Fig. 1). The identification of this strain was confirmed by NCBI sequence submission. Similarly, pectinolytic bacterial strains such as *Bacillus firmus*-I-4071, *B. firmus*-I-10104 and *Bacillus laterosporus*-I-107 were reported from agro and fruit processing industrial wastes (Geetha et al. 2012; Veeranjaneya Reddy et al. 2011; Kashyap et al. 2000).

### Optimization of media composition

The experimental results associated with the processing set of each independent variable (Table 1). To study the combined effects of these factors/variables, experiments were conducted at different combinations of these parameters using statistically designed experiments (Table 2). Experimental response along with predicted response was calculated from the regression equation for each run. A second order polynomial equation was derived to represent the pectinase production as a function of independent variables tested (Eq. 2).

**Table 1:** Actual and coded values of the media components in design

Factor	Name	Units	Low level	High level	Low coded	High coded	Mean	Std. dev
A	NaNO_3_	g/l	1.22	2.78	−1	1	2.00	0.43
B	KH_2_PO_4_	g/l	0.61	1.39	−1	1	1.00	0.21
C	KCl	g/l	0.34	0.66	−1	1	0.50	0.09
D	MgSO_4_·7H_2_O	g/l	0.34	0.66	−1	1	0.50	0.09
E	Yeast extract	g/l	0.61	1.39	−1	1	1.00	0.21
F	MIW powder	g/l	4.43	7.57	−1	1	6.00	0.85

Response	Name	Obs.	Analysis	Min.	Max.	Mean	Std. dev
R_1_	Pectinase activity (U/ml)	96	Polynomial	10.385	87.556	25.073	10.6414

*Std. Dev* standard deviation, *Obs* observations, *Min* minimum, *Max* maximum; A, B, C, D,and E represents the process parameters denoted as X_1_, X_2_, X_3_, X_4_, X_5_, and X_6,_ respectively for regression equation

**Table 2:** Experimental design with actual experimental and predicted value of responses of the central composite design

S. no	A	B	C	D	E	F	Actual pectinase U/ml	Predicted pectinase U/ml
1	0	0	0	0	0	0	22.29	27.70
2	−1	−1	−1	1	1	1	20.01	16.53
3	1	1	−1	−1	1	1	22.29	22.24
4	−1	1	1	−1	−1	1	17.14	22.69
5	1	−1	1	−1	1	−1	20.52	24.43
6	0	0	0	0	0	0	21.28	27.70
7	−1	−1	−1	−1	−1	−1	21.70	26.39
8	−1	1	1	1	1	−1	87.56	51.68
9	1	−1	1	1	−1	1	22.29	32.79
10	1	1	−1	1	−1	−1	21.95	24.87
11	1	−1	−1	−1	1	−1	14.10	15.75
12	0	0	0	0	0	0	27.02	22.54
13	−1	−1	1	1	1	1	33.69	28.67
14	1	1	1	1	−1	−1	20.35	23.30
15	1	−1	−1	1	−1	1	20.26	24.04
16	−1	−1	1	−1	−1	−1	13.59	11.05
17	1	1	1	−1	1	1	26.51	20.61
18	0	0	0	0	0	0	20.18	22.54
19	−1	1	−1	1	1	−1	24.40	29.22
20	−1	1	−1	−1	−1	1	25.33	27.71
21	1	1	−1	−1	−1	1	33.60	28.26
22	0	0	0	0	0	0	28.20	28.91
23	0	0	0	0	0	0	26.01	28.91
24	−1	1	1	1	−1	−1	55.98	43.99
25	1	−1	1	1	1	1	50.74	42.88
26	1	−1	1	−1	−1	−1	15.11	16.75
27	1	1	−1	1	1	−1	17.56	21.26
28	−1	−1	−1	−1	1	−1	17.48	22.78
29	−1	−1	−1	1	−1	1	24.15	22.56
30	−1	1	1	−1	1	1	20.26	32.79
31	0	0	0	0	0	0	18.15	22.43
32	−1	−1	1	−1	1	−1	19.59	19.82
33	−1	1	−1	−1	1	1	19.42	22.78
34	1	−1	−1	1	1	1	22.63	19.10
35	−1	1	−1	1	−1	−1	15.87	33.92
36	1	1	1	1	1	−1	14.78	32.07
37	−1	−1	1	1	−1	1	19.93	19.66
38	0	0	0	0	0	0	25.08	22.43
39	1	1	1	−1	−1	1	18.58	11.60
40	1	−1	−1	−1	−1	−1	50.24	20.45
41	0	0	0	0	0	0	23.81	24.28
42	1	−1	1	−1	1	1	27.19	28.85
43	−1	−1	−1	−1	−1	1	19.00	22.29
44	−1	1	1	−1	−1	−1	26.93	29.96
45	1	1	−1	1	−1	1	26.43	19.27
46	−1	1	1	1	1	1	41.63	37.56
47	1	1	−1	−1	1	−1	17.39	21.00
48	−1	−1	−1	1	1	−1	21.36	13.78
49	0	0	0	0	0	0	22.21	24.28
50	1	−1	1	1	−1	−1	16.89	21.53
51	1	−1	−1	1	−1	−1	21.78	16.68
52	1	−1	−1	−1	1	1	24.82	24.07
53	−1	1	−1	1	1	1	20.01	19.01
54	−1	1	−1	−1	−1	−1	48.63	38.88
55	1	1	1	1	−1	1	18.91	21.60
56	0	0	0	0	0	0	24.40	23.02
57	1	1	1	−1	1	−1	21.11	23.27
58	−1	−1	1	1	1	−1	12.33	29.82
59	−1	−1	1	−1	−1	1	19.25	10.86
60	0	0	0	0	0	0	19.00	23.02
61	−1	−1	−1	1	−1	−1	14.18	19.58
62	1	−1	1	−1	−1	1	14.02	20.95
63	0	0	0	0	0	0	30.65	25.26
64	1	1	−1	−1	−1	−1	22.21	26.80
65	−1	1	1	−1	1	−1	39.85	39.83
66	−1	−1	−1	−1	1	1	23.05	18.46
67	0	0	0	0	0	0	24.74	25.26
68	−1	1	1	1	−1	1	22.21	29.65
69	1	1	−1	1	1	1	24.23	15.44
70	1	−1	1	1	1	−1	37.49	31.40
71	−1	1	−1	−1	1	−1	29.47	36.15
72	−1	−1	1	−1	1	1	23.56	21.82
73	0	0	0	0	0	0	33.69	25.10
74	0	0	0	0	0	0	48.38	25.10
75	1	−1	−1	1	1	−1	10.39	13.94
76	1	−1	−1	−1	−1	1	12.50	30.96
77	1	1	1	1	1	1	21.36	32.57
78	−1	1	−1	1	−1	1	22.37	25.90
79	−1	−1	1	1	−1	−1	21.53	23.01
80	1	1	1	−1	−1	−1	27.78	16.46
81	−1.565	0	0	0	0	0	22.80	28.44
82	0	0	0	0	0	0	26.43	25.91
83	1.565	0	0	0	0	0	18.91	23.38
84	0	0	0	0	−1.565	0	27.27	24.32
85	0	0	0	0	1.565	0	23.47	27.50
86	0	−1.565	0	0	0	0	18.07	21.74
87	0	0	0	0	0	0	26.68	25.91
88	0	0	0	0	0	0	28.20	25.91
89	0	0	−1.565	0	0	0	26.34	23.13
90	0	0	0	1.565	0	0	34.36	27.88
91	0	0	0	0	0	−1.565	21.70	27.03
92	0	0	0	0	0	1.565	30.40	24.79
93	0	0	0	0	0	0	27.27	25.91
94	0	0	0	−1.565	0	0	25.58	23.94
95	0	0	1.565	0	0	0	28.12	28.69
96	0	1.565	0	0	0	0	28.96	30.09

### Experimental design

#### Statistical analysis

The ANOVA result of quadratic regression model for pectinase yield is described in Table 3.

**Table 3:** Analysis of variance table (partial sum of squares-type III)

Source	Sum of squares	*df*	Mean square	*F* value	*p* value Prob > F
Block	410.20	8	51.28		
Model	4052.25	11	368.39	4.45	<0.0001 significant
A-NaNO_3_	180.55	1	180.55	2.18	0.1440
B-KH_2_PO_4_	490.32	1	490.32	5.92	0.0173
C-KCl	218.10	1	218.10	2.63	0.1088
D-MgSO_4_·7H_2_O	109.29	1	109.29	1.32	0.2543
E-Yeast extract	71.38	1	71.38	0.86	0.3562
F-MIW powder	35.02	1	35.02	0.42	0.5175
AB	746.63	1	746.63	9.01	0.0036
AF	289.81	1	289.81	3.50	0.0653
BF	401.69	1	401.69	4.85	0.0307
CD	758.21	1	758.21	9.15	0.0034
CE	751.25	1	751.25	9.07	0.0035
Residual	6295.24	76	82.83		
Lack of fit	6101.84	65	93.87	5.34	0.0022 significant
Pure error	193.41	11	17.58		
Cor total	10757.6	95			

Equation:

2 $$ \begin{aligned} {\text{Pectinase}}\,\, ( {\text{U/ml)}}  \,  = + 28.25 - 1.62 \times A + 2.67 \times B + 1.78 \times C + 1.26 \times D + 1.02 \times E \, - 0.71 \times F - 3.42 \times A \times B \\\ - 1.52 \times A \times C - 1.14 \times A \times D - 0.87 \times A \times E + 2.13 \times A \times F \\  + 0.93 \times B \times C + 0.080 \times B \times D - 0.45 \times B \times E - 2.51 \times B \times F + 3.44 \times C \times D \\  + 3.43 \times C \times E - 0.71 \times C \times F + 1.78 \times D \times E + 0.87 \times D \times F + 1.48 \times E \times F \\  - 2.52 \times A^{2} - 1.44 \times B^{2} + 0.081 \times C_{2} + 1.20 \times D_{2} - 0.68 \times E_{2} - 0.40 \times F_{2} \\ \end{aligned} $$

The statistical significance of Eq. (2) was verified by the ‘*F-*test and the ‘analysis of variance’ for the quadratic model of the response surface (Table 3). The Model F-Valueof 1.74 implies that the model is significant. There is only a 4.66 % chance that a “Model F-Value” this large could occur due to noise. Values of “Prob > F” less than 0.0500 indicate model terms are significant. ANOVA (*F-*test) for the model explained the response of the dependent variable ‘*Y*’. In this case B, AB, BF, CD and CE are significant model terms. If there are many insignificant model terms (not counting those required to support hierarchy), model reduction may improve model. Values greater than 0.1000 indicate the model terms are not significant. The *R* ^2^ which can be defined as the ratio of the explained variation to the total variation was a measure of the degree of fit. The closer the *R* ^2^ value to unity, the better the empirical model fits the actual data. The value of determination of coefficient *R* ^2^ is 0.7523, which indicated that the model could explain 75.23 % of variability and is unable to explain only 24.77 % of the total variation. The closer the value of *R* ^2^ to 1 indicates the better correlation between the observed and predicted values suggesting a good fit for submerged fermentation.

**Fig. 2:**
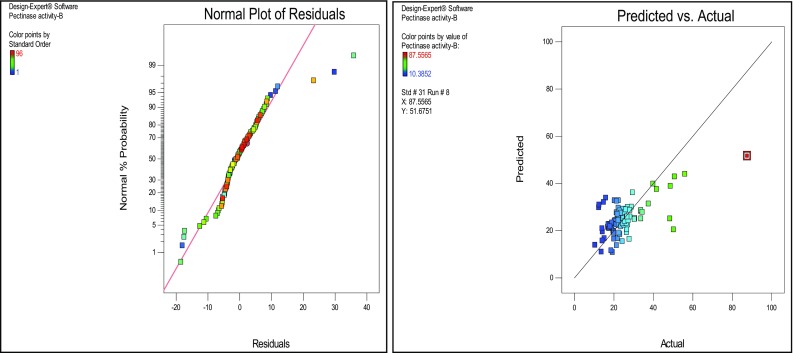
*Plots* of normal probability % and correlation between actual and predicted values

**Fig. 3:**
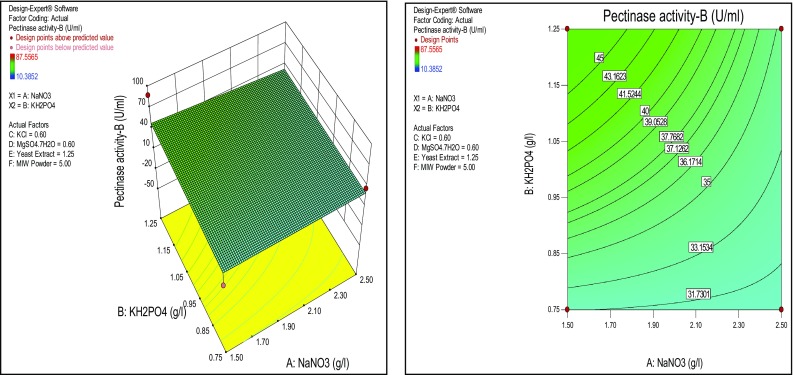
Response surface *plots* of effect of NaNO_3_ and KH_2_PO_4_ on pectinase production

**Fig. 4:**
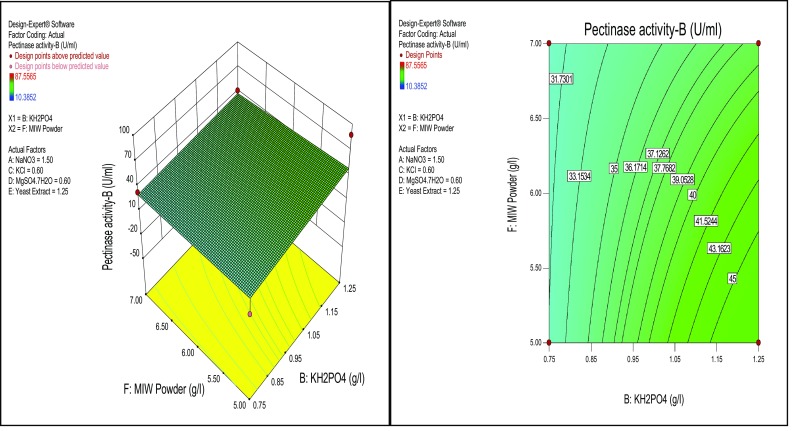
Response surface *plots* of effect of KH_2_PO_4_ and MIW powder on pectinase production

The adjusted *R* ^2^ was a corrected value for *R* ^2^ after elimination of the unnecessary model terms. If many non-significant terms have been included in the model, the adjusted *R* ^2^ would be remarkably smaller than the *R* ^2^. The adjusted *R* ^2^ was 0.3663, which is more suitable for comparing models with different numbers of independent variables. The coefficient of variation (CV) is a measure of residual variation of the data relative to the size of the mean; the small values of CV give better reproducibility. A lower value for the CV 36.30 % clearly indicates high degree of precision and higher reliability of the experimental values. The significance of individual variables can be evaluated from their *p* values, the more significant terms having a lower *p-*value. The *p-*values are used to check the significance of each coefficient which also indicates the interaction strength between each independent variable. The *p-*values of each of the variables also indicate their quadratic and interaction terms. Besides the relationship between the actual experimental and predicted values, the plotted points cluster around the diagonal line, indicating good fitness of the model and they are in reasonable agreement (Fig. 2). An effect of concentration of KH_2_PO_4_ has shown significance on the pectinase production than NaNO_3_ and MIW powder was analyzed by the response surface plots (Figs. 3, 4).

**Fig. 5:**
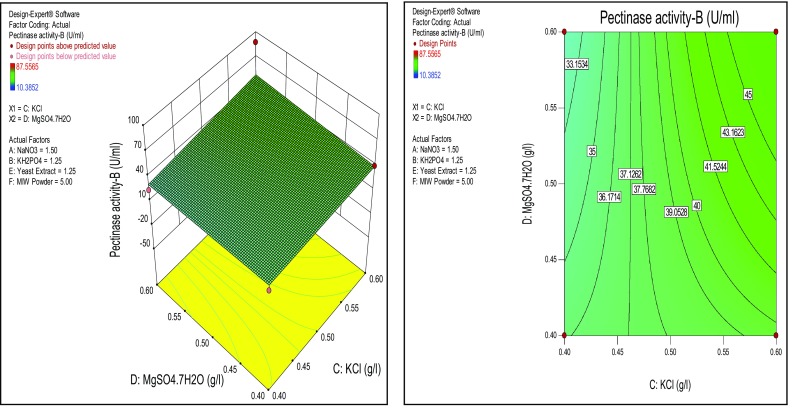
Response surface plots of effect of KCl and MIW powder on pectinase production

**Fig. 6:**
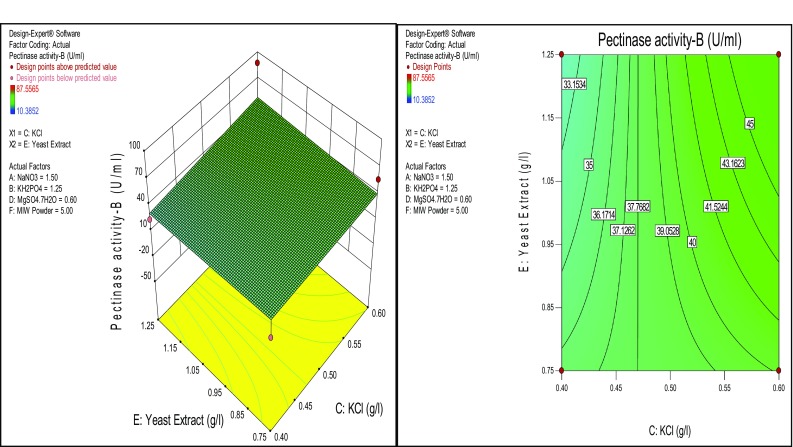
Response surface *plots* of effect of KCl and Yeast extract on pectinase production

Similarly, KCl has shown significance on the pectinase production than yeast extract and MgSO_4_·7H_2_O, respectively (Figs. 5, 6). All the response surfaces could be analyzed for determining the optimized value of the factors. Enzyme production is a growing field of biotechnology, the majority of the industrial enzymes are of microbial origin. Though there is a conventional method for the optimization of media composition RSM is the best statistical method to optimise all media components at a time. Through this method we can find out the suitable medium with optimal cultural conditions for high production of microbial pectinase by submerged fermentation. In the present study, the *Enterobacter* sp. PSTB-1 showed pectinase activity ranging from 10.3852 U/ml (low) to 87.5565 U/ml (high) under RSM designed conditions. The highest activity 87.5565 U/ml of *Enterobacter* sp. PSTB-1 is higher than the pectinase activity 53 U/ml of *Bacillus* sp. DT7 and 10.65 U/ml of *Bacillus licheniformis* (Shahera et al. 2002; Hou et al. 2011).

## Conclusion

Model summary statistics showed that the predicted model was in close agreement with the experimental data. From the ANOVA results, the second-order polynomial regression model developed has a high correlation *R* ^2^ value. The best conditions were found to be, NaNO_3_ 2.0 g/l, KCl 0.50 g/l, KH_2_PO_4_ 1.0 g/l, MgSO_4_·7H_2_O 0.50 g/l, Yeast extract 1.0 g/l, Mango industrial waste powder 5.0 g/l. Under these optimized conditions, the experimental value 87.557 U/ml of enzyme production closely agreed with the predicted value 53.658/ml. This is the first report regarding pectinase production from *Enterobacter* sp. PSTB-1 by using mango fruit processing industrial waste as a carbon source. The results resembled that the followed design model of response surface methodology (RSM) is the best fit for conducting the experiments to know the required concentrations of media components for the high production of pectinase. It is the cheapest fermentation process for high production of industrially important and microbial derived pectinase.
